# MiR-23a-mediated inhibition of topoisomerase 1 expression potentiates cell response to etoposide in human hepatocellular carcinoma

**DOI:** 10.1186/1476-4598-12-119

**Published:** 2013-10-08

**Authors:** Ning Wang, Meifen Zhu, Sai-Wah Tsao, Kwan Man, Zhangjin Zhang, Yibin Feng

**Affiliations:** 1 School of Chinese Medicine, Li Ka Shing Faculty of Medicine, The University of Hong Kong, 10 Sassoon Road, Pokfulam, Hong Kong, PR China; 2 Department of Anatomy, Li Ka Shing Faculty of Medicine, The University of Hong Kong, Hong Kong, PR China; 3 Department of Surgery, Li Ka Shing Faculty of Medicine, The University of Hong Kong, Hong Kong, PR China

**Keywords:** miR-23a, Topoisomerase 1, Etoposide, Hepatocellular carcinoma, DNA damage

## Abstract

**Background:**

microRNAs have been shown to regulate the chemosensitivity of cancer cells. The aim of this study is to investigate the role and mechanism of mir-23a in enhancing the anti-tumor effect of topoisomerase 2A (TOP2A) poison etoposide in human hepatocellular carcinoma (HCC).

**Methods:**

The anti-tumor effect of chemotherapeutic agents in HCC cells were examined in vitro and in vivo xenograft model. Expression of mRNA and miRNAs were determined by quantitative real-time PCR. Protein expression was analyzed by immunoblotting.

**Results:**

Overexpression of mir-23a could significantly potentiate the in vitro and in vivo anti-tumor effect of etoposide; however, ectopic expression of miR-23a fails to sensitize HCC cells to 5-fluorouracil treatment, indicating the miR-23a-induced cancer cell hypersensitivity in chemotherapy is TOP2A-specific though miR-23a overexpression could not directly up-regulate TOP2A expression. Topoisomerase 1(TOP1) is down-regulated in miR-23a-overexpressed HCC cells. MiR-23a could directly bind to 3′untranslated region of TOP1 mRNA, and suppress the corresponding protein expression and inhibition of miR-23a further arguments the expression of TOP1. MiR-23a was up-regulated during DNA damage in cancer cells in line with the p53 expression. Up-regulation of p53 induces mir-23a expression, while suppression of p53 inhibits miR-23a in HCC cells.

**Conclusion:**

Our study sheds light on the role of miR-23a as a potential target in regulating chemosensitivity of HCC cells.

## Introduction

MicroRNAs (miRNAs) are small non-coding RNAs with an average length of 20–22 nucleotides. Recent studies have revealed that miRNAs are involved in many different physiological and pathological processes via regulating functional genes’ expression [1]. With direct binding to the 3′-untranslated region (3′-UTR) of these mRNAs, one individual miRNA could regulate hundreds of genes via inducing mRNA degradation or prohibiting gene translation [2,3]. Accumulating evidences have shown that expression profile of miRNAs differs from that in normal human tissue [4]. The differential expression of miRNAs in consequence regulates oncogenic factors or tumor suppressors, leading to onset/offset of angiogenesis, growth and metastasis of human cancers [5].

It was further noticed that miRNAs play critical roles in regulating tumor cell response to chemotherapeutic agents. Previous studies have shown that the expression profile of miRNAs in chemoresistant human cancer cell lines varies from chemo-responsive tumor cells [6]. Altered expression of miRNAs could control the chemosensitivity of cancer cells by, either directly regulating apoptosis-related Bcl-2 family proteins to modify the cellular response to apoptosis initiated by chemotherapeutic agents, or modulating drug availability to influence responsiveness of tumor cells indirectly [7]. TOP2A is one of cellular topoisomerases that determines tumor cell response to chemotherapeutics. A recent study found that chemosensitivity of tumor cells could be determined by intracellular topoisomerase level [8], which provides a novel strategy in enhancing drug response in cancer therapy. Discovered as a Topoisomerase 2A (TOP2A) poison, etoposide is now a frontline chemotherapeutics in treating various human cancers [9]. However, the mechanism of different responses of tumor cells to etoposide is not yet clear. A recent study shows that expression of miRNAs varies in etoposide resistant breast cancer cells [10], but how miRNAs regulate cell response upon etoposide remains unclear.

In this study, we report that overexpression of miR-23a could sensitize HCC to the in vitro and in vivo treatment of etoposide. MiR-23a fails to boost up response of HCC cells to 5-fluorouracil (5-Fu) treatment, indicating that the regulation of miR-23a on response of HCC cell may be TOP2A poisons-specific. Overexpression of miR-23a could further impair the cell progression through S phase when HCC cells were exposed to etoposide, while the TOP2A expression has not changed. The other topoisomerase, TOP1 was suppressed in miR-23a-overxpressed HCC cells and was validated as the direct target of miR-23a. Suppression of TOP1 expression by miR-23a results in reduction of overall intracellular topoisomerase activity when the cells are exposed to etoposide, which in consequence enhances drug response of HCC cells. Forced overexpression of miR-23a in HCC cells reduces the cytotoxicity of TOP1 poison irinotecan, and up-regulation of miR-23a could be observed in HCC cells upon DNA damage, during which miR-23a may play a role in the intracellular TOP1 homeostasis. Furthermore, we found expression of miR-23a is regulated by p53 in HCC cells. Our findings shed light on the role of miR-23a as a potential target in regulating drug responses of HCC cells.

## Materials and methods

### Plasmids and siRNA

The pCMV-MIR Vectors with and without human miR-23a expression, luciferase expressing-pMir-Target Vectors with and without TOP1 3′UTR expression were purchased from Origene (USA). The pRL-CMV *Renilla Luciferase* (Ruc) Control Reporter Vectors were obtained from Promega (USA). The siRNA against human p53 were from Santa Cruz (USA). The scramble negative control to miRNAs (scr negative control) and inhibitor against miR-23a were purchased from Exiqon (Denmark).

### Cell line and cell culture

The human hepatocellular carcinoma cells HepG2 and embryonic kidney cell line HEK293T were obtained from American Type Culture Collection (ATCC, USA). MHCC97L cell line was kindly gifted by Dr. Man Kwan from Department of Surgery, The University of Hong Kong and has been used in our previous published studies [11,12]. Cells were cultured in High Glucose Dulbecco’s Modified Eagle Medium supplemented with 10% Fetal Bovine Serum and 1% Penicillin/Streptomycin.

### Cell viability assays

The cell viability was determined by MTT assay. Briefly, cells were seeded into 96-well cell culture plate and received treatments. 4 h before the end of experiment, 10 μl of 3- (4,5-cimethylthiazol-2-yl)-2,5-diphenyl tetrazolium bromide (5 mg/mL) was added and cells were incubated at 37°C. Then medium was removed and the residue was dissolved in DMSO. The absorbance of each well was read at 570 nm with a microplate reader.

### Irradiation

Cells were exposed to UV-C irradiation (254 nm) for 10 min then incubated at 37°C, 5% CO_2_.

### Quantitative real-time PCR

*Detection of mRNA* Total RNA was extracted with Total RNA purification kit (Norgan, Canada). The Taqman® Gene Expression Assay (Hs00243257_m1) was conducted for the detection of TOP1 mRNA transcripts with LigherCycler480 (Roche, USA). GAPDH (Hs02758991_g1) was used as the loading control. For detection of TOP2A, SYBR Green I qPCR assay was conducted with primers (*left: GCGAGTGTGCTGGTCACTAA; right: ACAATTGGCCGCTAAACTTG*). GAPDH was used as the loading control *(left: GAGTCCACTGGCGTCTTCAC; right: TTCACACCCATGACGAACAT)*.

*Detection of miRNAs* The total RNA containing miRNAs were extracted with miRNeasy mini Kit (Qiagen, Germany) under the manufacturer’s instruction. The Taqman® MicroRNA Assay (000399) was conducted for the detection of mature miR-23a with U6 as loading control (001973); The Taqman® Pri-miRNA Assay (Hs04270764_pri) was conducted to detect the expression of pri-miR-23a with GAPDH as loading control (Hs02758991_g1); For the detection of pre-miR-23a, SYBR Green I qPCR Assay was conducted with primers *(left: CTGGGGATGGGATTTGCT; right: TGGAAATCCCTGGCAATG)*. GAPDH was used as the loading control *(left: GAGTCCACTGGCGTCTTCAC; right: TTCACACCCATGACGAACAT)*.

### Immunoblotting

Cells were lysed with Radioimmunoprecipitation assay (RIPA) buffer with complex cocktailed proteinase inhibitor (Roche, USA) and phosphatase inhibitor (1 mM Na_3_VO_4_ and 1mM NaF) on ice for 30 minutes followed by centrifugation at 14,000 rpm at 4°C for 15 minutes. Supernatants were transferred and protein concentrations were determined by BSA assay (Bio-rad, USA). Equal yields of protein were separated on SDS-PAGE and transferred onto a polyvinylidene fluoride membrane (PVDF, Biorad). The membrane was then blocked in buffer containing 5% BSA, Tris (10 mmol/L, pH7.4), NaCl (150 mmol/L) and Tween 20 (1%) at room temperature for one hour with gentle shaking. The membrane was then incubated with primary antibodies at 4°C overnight followed by incubation with appropriate secondary antibody (Abcam, UK) at room temperature for 1 hour. The immunoreactivites were detected using ECL advanced kit (GE Healthcare, UK) and visualized using a chemiluminenescence imaging system (Bio-Rad, USA).

### Cell cycle analysis

Cells were seeded in 6-well plate with 50% confluence and were synchronized by overnight starvation. After treatment the cells were collected and fixed in 75% alcohol for 24 h. Then cell pellet was stained with 50 μg/mL Propidium iodide (PI) in PBS for 40 min in dark at room temperature. Cells were washed and resuspended and the cell cycle distribution was determined with Flow cytometer (BD FACSCantoII Analyzer, USA). The raw data was collected and analyzed by Flow Jo 7.6 software (USA).

### Luciferase assay

The HEK293T cells were co-transfected with pMIR Vectors containing TOP1 3′UTR and miR-23a mimics. Cells were lysed and the Firefly luciferase activity was detected and *Renilla* lucfierase activity was used for normalization. The lysate was detected with Dual Luciferase reporter assay kit (Promega, USA) with a luminometer.

### Xenograft model

The tumor xenograft model was used in this study. Briefly, 6-week-old female BALB/c nu/nu athymic nude mice received MHCC97L cells transfected with pCMV vector injection subcutaneously in 0.2 ml at its left side of waist and MHCC97L cells with miR-23a overexpression injection at its right. Mice was then randomized into different groups (n = 5). Etoposide treatment group received 0.2 mL i.p. injection of 7.5 mg/kg etoposide, while 5-Fu treatment group received the same volume of 25 mg/kg 5-Fu. Control group received the same volume of PBS. Tumor volume and body weight were measured 3 times per week for 4 weeks. At the end of the experiment, the mice were sacrificed with overdose of Phenobarbital (200 mg/kg) and the tumor was dissected out. All animals received human care and study protocols complied with the guidelines of the Laboratory Animal Centre of The University of Hong Kong and were approved by the Committee on the Use of Live Animals in Teaching and Research (CULATR) of the University of Hong Kong. Moreover, animals were processed according to the suggested international ethical guidelines for the care of laboratory animals throughout the experiments.

### Statistical analysis

Results were analyzed using student T-test and expressed as mean ± SD.

## Results

### Overexpression of miR-23a sensitizes tumor cell to TOP2A poisons

**Figure 1:**
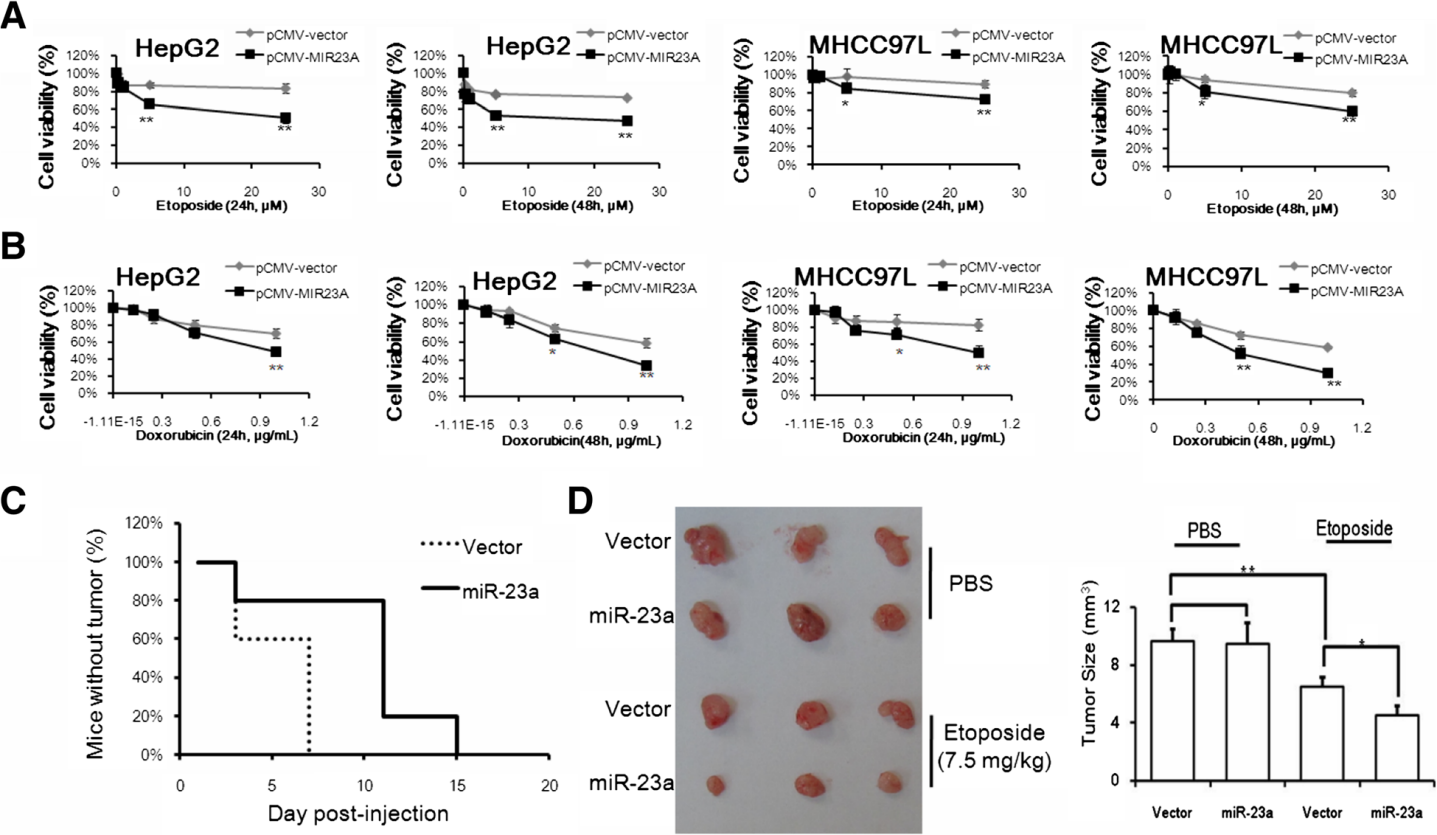
**miR-23a enhances cytoxicity of etoposide in human HCC. A** shows etoposide exhibits more toxic to HCC cells with ectopic miR-23a expression. HepG2 and MHCC97L cells with or without expressing ectopic miR-23a were treated with etoposide for 24 and 48 h. Cytotoxicity was evaluated with MTT assay. Increasing cell death after etoposide treatment was observed in miR-23a-overexpressed HCC cells; **B** shows ectopic miR-23a could significantly potentiate HCC cells to doxorubicin treatment. HepG2 and MHCC97L cells with or without expressing ectopic miR-23a were treated with doxorubicin for 24 and 48 h. Cytotoxicity was evaluated with MTT assay. **C** shows that miR-23a overexpression enhances inhibition of HCC tumorigenesis by etoposide. Mice were subcutaneously injected with MHCC97L cells with or without ectopic miR-23a and treated with etoposide (25 mg/kg/2 days, i.p.). Megascopic xenograft was recorded. Delayed presence of megascopic tumor was observed in miR-23a-expressed group. **D** shows that miR-23 overexpression increase tumor response to etoposide. After treatment for 3 weeks, tumor was dissected out and calculated. Significant reduction on tumor size could be found in miR-23a-expressed group. This picture presents the 3 representatives in each group.

A growing body of studies has focused on the regulation of cancer cell’s response to chemotherapeutics by miRNAs [13]. The role of miRNAs in cancer therapy was further evidenced with the observation that HCC patients with lower miR-26 level has shorter time of survival but better response to interferon therapy though miR-26 is highly expressed in HCC in compared with non-tumor hepatic tissues [14]. Although it was found the expression of miR-23a is slightly increased in HCC as evidenced by literature [15] and our own study (Additional file 1: Figure S1), we found that overexpression of miR-23a could potentiate the response of HCC to TOP2A poison etoposide (Figure 1A). This was further evidenced by the fact that HCC cells with ectopic miR-23a are vulnerable to another TOP2A poison doxorubicin (Figure 1B). In vivo study shows that overexpression of miR-23a could promote the tumor-free survival of the mice upon treatment of 7.5 mg/kg etoposide (Figure 1C). Tumor size was significantly reduced by ectopic miR-23a in mice treated with etoposide (Figure 1D). These findings suggest that high level of miR-23a may particularly potentiate cellular response to TOP2A poisons.

### TOP2A is responsible for the hypersensitivity of miR-23a-overxpressing HCC cells to etoposide

**Figure 2:**
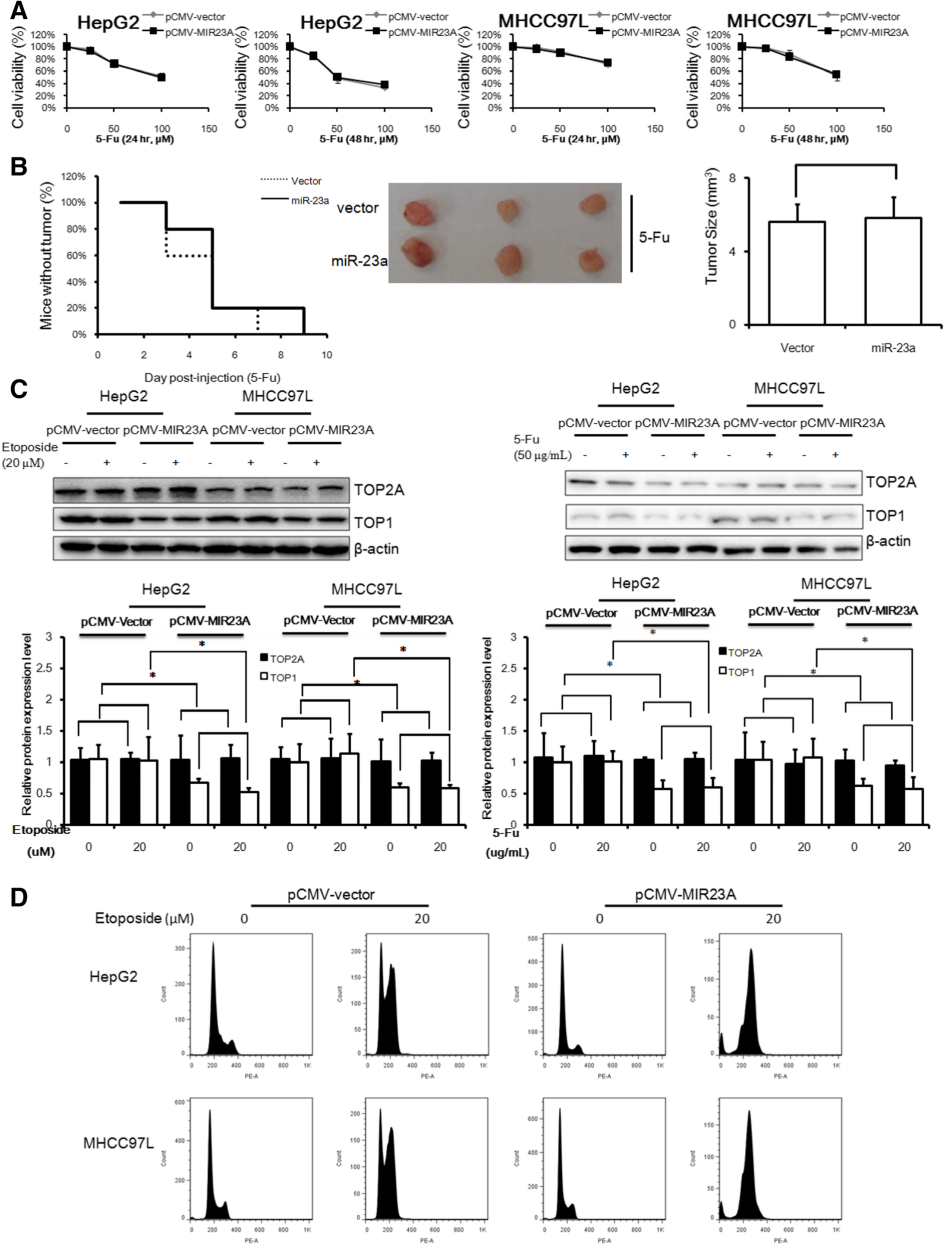
**miR-23a-induced HCC cell hypersensitivity to 5-Fu treatment requires TOP2A. A** shows that miR-23a fails to potentiate HCC cells to 5-Fu treatment. HepG2 and MHCC97L cells with or without expressing ectopic miR-23a were treated with etoposide for 24 and 48 h. Cytotoxicity was evaluated with MTT assay. No different cytotoxicity was observed in miR-23a-overexpressed cells in compared with wild-type cells. **B** shows that miR-23a could not enhance chemotoxocity of 5-Fu in vivo. Methodologies were described as Figure 1C and D and mice were treated with 5-Fu (25 mg/kg/2 days, i.p.). Neither delayed presence of megascopic xenograft nor reduced tumor size could be observed in miR-23a-overexpressed group. This picture presents the 3 representatives in each group. **C** shows that miR-23a suppresses TOP1 expression without significantly inducing TOP2A in HCC cells. Wild-type and miR-23a-overexpressed HCC cells were treated with etoposide (20 μM) or 5-Fu (50 μg/mL) for 24 h then protein was collected. The expression of TOP1 and TOP2A was analyzed with immunoblotting. Significant reduction of TOP1 expression in miR-23a overexpressed HCC cells could be observed. No potent up-regulation of TOP2A could be found. **D** shows that overexpression of miR-23a further impaired cell progression through S phase in etoposide-treated HCC cells. Wildtype and miR-23a-overexpressed HCC cells were treated with etoposide (20 μM) for 24 h and then fixed. Cells were then stained with PI for cell cycle analysis. Accumulation at S phase was observed after etoposide treatment and ectopic miR-23a enhances this effect of etoposide. *p < 0.05; p < 0.01 in comparison.

As the drug response of miR-23a-overexpressing HCC cells was potentiated particularly when cells were treated with TOP2A poisons, we assumed that TOP2A is required for miR-23a-mediated increased chemosensitivity in HCC cells. To test this assumption, we treated the HCC cells with 5-Fu. No significant difference could be observed between the cytotoxicity of 5-Fu on wild-type or miR-23a-overexpressing HCC cells (Figure 2A). In vivo study further confirmed that forced miR-23a expression could not potentiate the response of HCC xenograft to 5-Fu (25 mg/kg/2 days). Neither delay on tumorigenesis nor reduced end-point tumor size could be found in miR-23-overexpressed xenograft in compared with wild-type (Figure 2B). These data suggest that the hypersensitivity mechanism may be TOP2A poisons specific. Previous studies have shown that the chemosensitivity of TOP2A poisons could be regulated by the expression level of TOP2 protein in cancer cells [16]. To further decipher the role of TOP2A in miR-23a-regulated chemosensitivity of HCC cells to TOP2A poison, we first confirmed if the cellular expression of TOP2A has been altered. The protein expression of TOP2A remains unchanged with miR-23a was forcedly expressed in HCC cells, while TOP1 was significantly suppressed in miR-23a-overexpressed HCC cells (Figure 2C). Previous study shows that tumor cells were sensitive to TOP2A poisons without initiating compensatory expression of TOP2A when TOP1 was suppressed [8], indicating that increased response to etoposide may be independent to TOP2A expression. Cellular response to etoposide when TOP1 was suppressed by miR-23a may be indicated by the increasing impaired cell progression through S phase upon etoposide treatment (Figure 2D), and interestingly, cell cycle progression of wild-type and miR-23a-overexpressed HCC cells with treatment of 5-Fu have no differences (Additional file 1: Figure S2) These data suggest that simultaneous down-regulation of TOP1-mediated enhanced response to etoposide may be responsible for the enhancing sensitivity to TOP2A poison in HCC cells with miR-23a overexpression.

### TOP1 is the direct target of miR-23a in hepatocellular carcinoma

**Figure 3:**
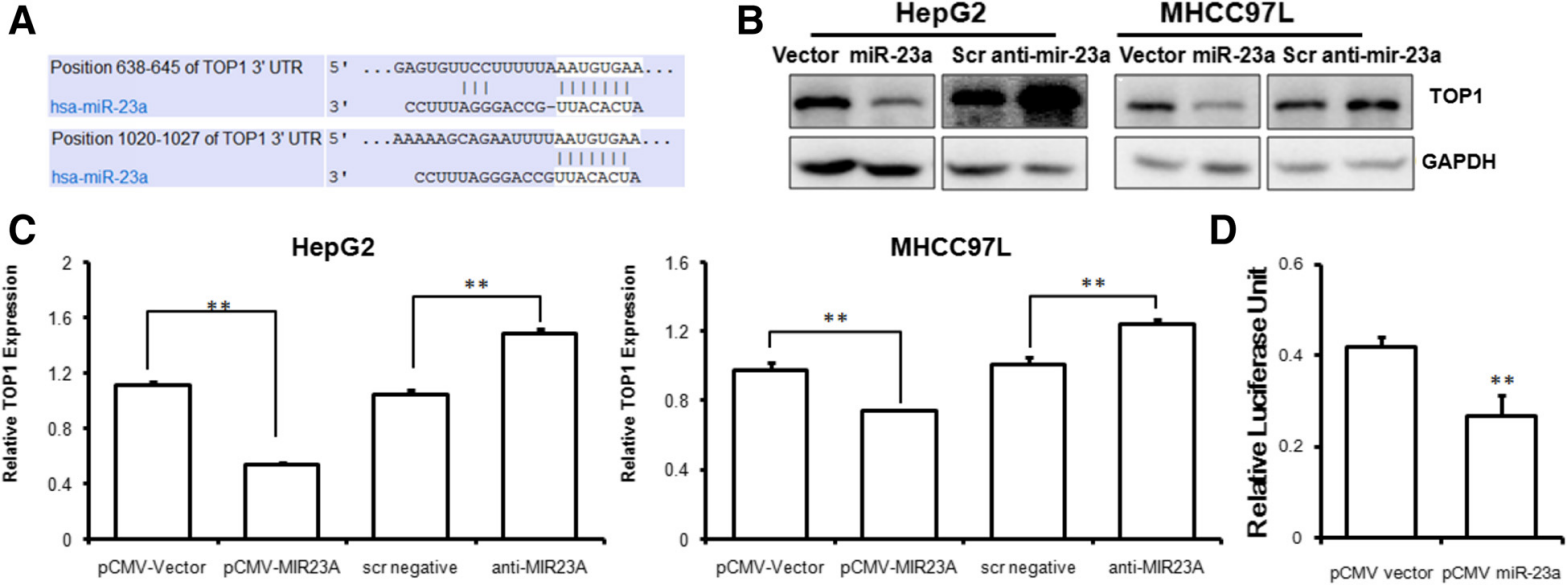
**TOP1 is the direct target of miR-23a. A** shows that the possible target of TOP1 3′UTR interaction with miR-23a predicted by TargetScan software. **B** shows that TOP1 protein expression was inhibited by miR-23a. The cells were transiently transfected with either vector of miR-23a-expressing plasmids. Significant suppression of TOP1 protein level could be observed in miR-23a-overexpressing cells; **C** shows that TOP1 mRNA transcript was inhibited after long exposure to miR-23a. The cells were transiently transfected with either vector of miR-23a-expressing plasmids. Potent inhibition of TOP1 mRNA transcripts could be observed in cells with exposure to miR-23a. **D** shows that the luciferase activity of vector expressing TOP1 3′UTR could be suppressed in the presence of miR-23a. HEK293T cells were co-transfected with luciferase-containing plasmid expressing TOP1 3′UTR and the plasmid expressing miR-23a. The luciferase activity was detected by dual luciferease reporting assay. Activity of *Renilla Luciferase* was expressed as control.

The possible target of miR-23a was predicted with TargetScan5.2 software, and miR-23a was predicted to directly bind to the 3′UTR region of human TOP1 mRNA (Figure 3A). Immunoblotting assay confirmed the inhibition of TOP1 expression by miR-23a (Figure 3B), which was in line with the mRNA inhibition of TOP1 by miR-23a (Figure 3C). Suppression of miR-23a by specific inhibitor restored the mRNA and protein expression of TOP1 (Figure 3B and C). Significant inhibition of TOP1 mRNA by miR-23a overexpression indicated a post-transcriptional regulatory mechanism is involved [17]. We then conducted luciferase reporter assay to confirm the direct binding of miR-23a to the 3′UTR of TOP1 mRNA. With co-transfection of the luciferase reporter vector encoding 3′UTR of TOP1 mRNA and miR-23a into HE293T cells, we observed that the luciferase activity was significantly suppressed by miR-23a transfection (Figure 3D). These results indicate that TOP1 may be the direct target of miR-23a.

### DNA damage-induced miR-23a regulates TOP1 expression in hepatoceullar carcinoma cells

**Figure 4:**
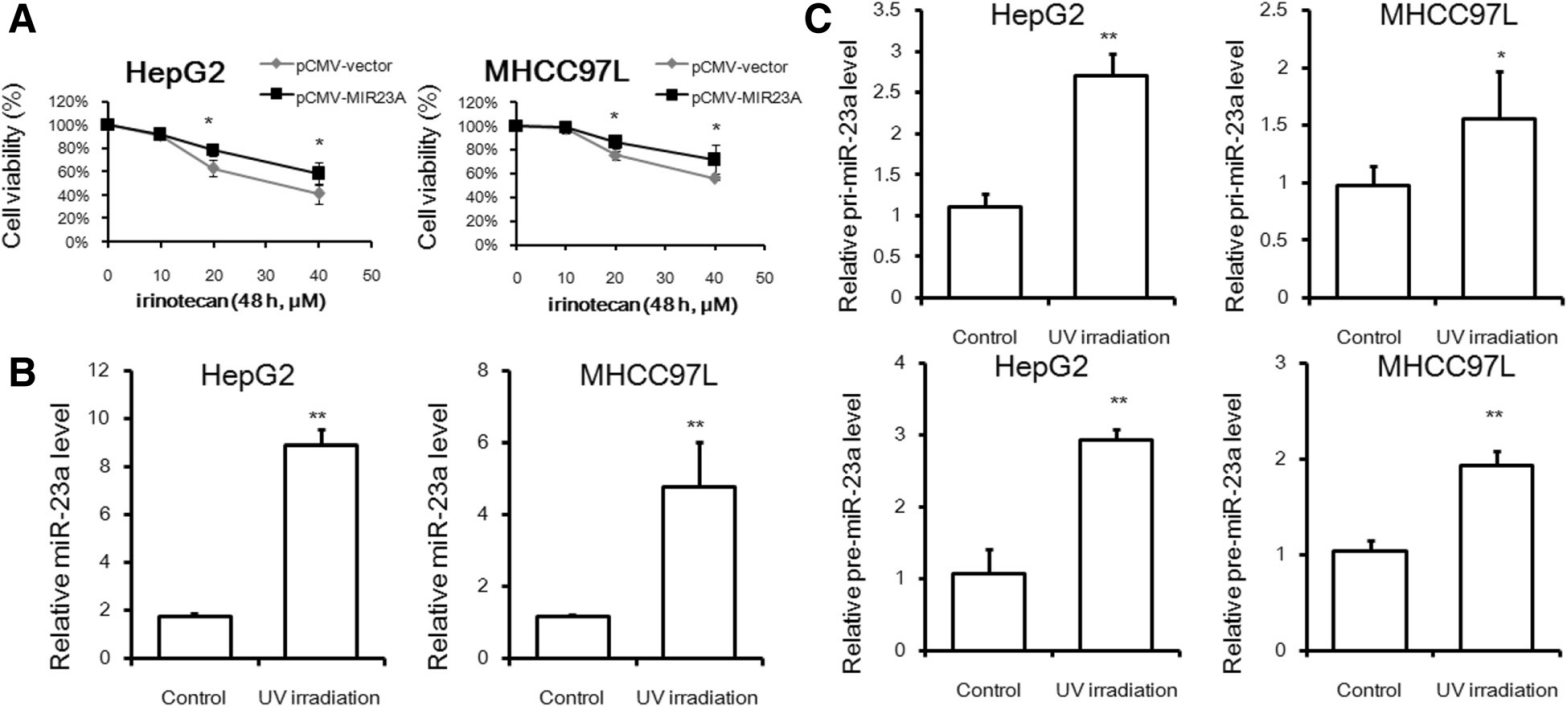
**DNA damage-induced miR-23a regulates TOP1 expression. A** shows that overexpression of miR-23a attenuates the cytoxicity of TOP1 poison irinotecan. Wild-type and miR-23a overexpressed HCC cells were treated with irinotecan and cell viability was determined by MTT assay. **B** shows that miR-23a was up-regulated upon DNA damage. Cells were exposed to UV-C for 10 min followed by 24 h culture. Total RNA was collected and miR-23a was detected by RT-qPCR. **C** shows miR-23a was transcriptionally activated upon DNA damage; RNA was collected and the pri-miR-23a and pre-miR-23a were detected as described.

The interaction between miR-23a and TOP1 was further evidenced in our study by the fact that forced overexpression of miR-23a significantly reduced the response of HCC cells with TOP1 poison irinotecan treatment (Figure 4A). As the content of TOP1 could predominantly regulate sensitivity of cancer cells in response to TOP1 poisons, suppression of TOP1 expression by miR-23a is therefore capable to reduce the content of TOP1-DNA covalent complex through removing available TOP1 from the system. Since TOP1 is critically required for DNA repair, we examined if miR-23a could be regulated during DNA damage/repair which may further evidence the regulation of TOP1 expression by miR-23a. Interestingly, induced expression of miR-23a in hepatoma cells could be also observed upon DNA damage induced by either UV irradiation or hydrogenperoxide treatment (Figure 4B and Additional file 1: Figure S3), which suggested that miR-23a up-regulation might be the common feature during DNA damage. Initiation of pri- and pre-miR-23a expressions in hepatoma cells exposed to UV irradiation exhibits that miR-23a was transcriptionally activated upon DNA damage (Figure 4C). In addition, we observed that the expression of p53, one of the most commonly transcription activator of miRNAs, was induced during DNA damage (Additional file 1: Figure S4), which was consistent with previsous report [18].

### Expression of miR-23a was transcriptional activated by p53

**Figure 5:**
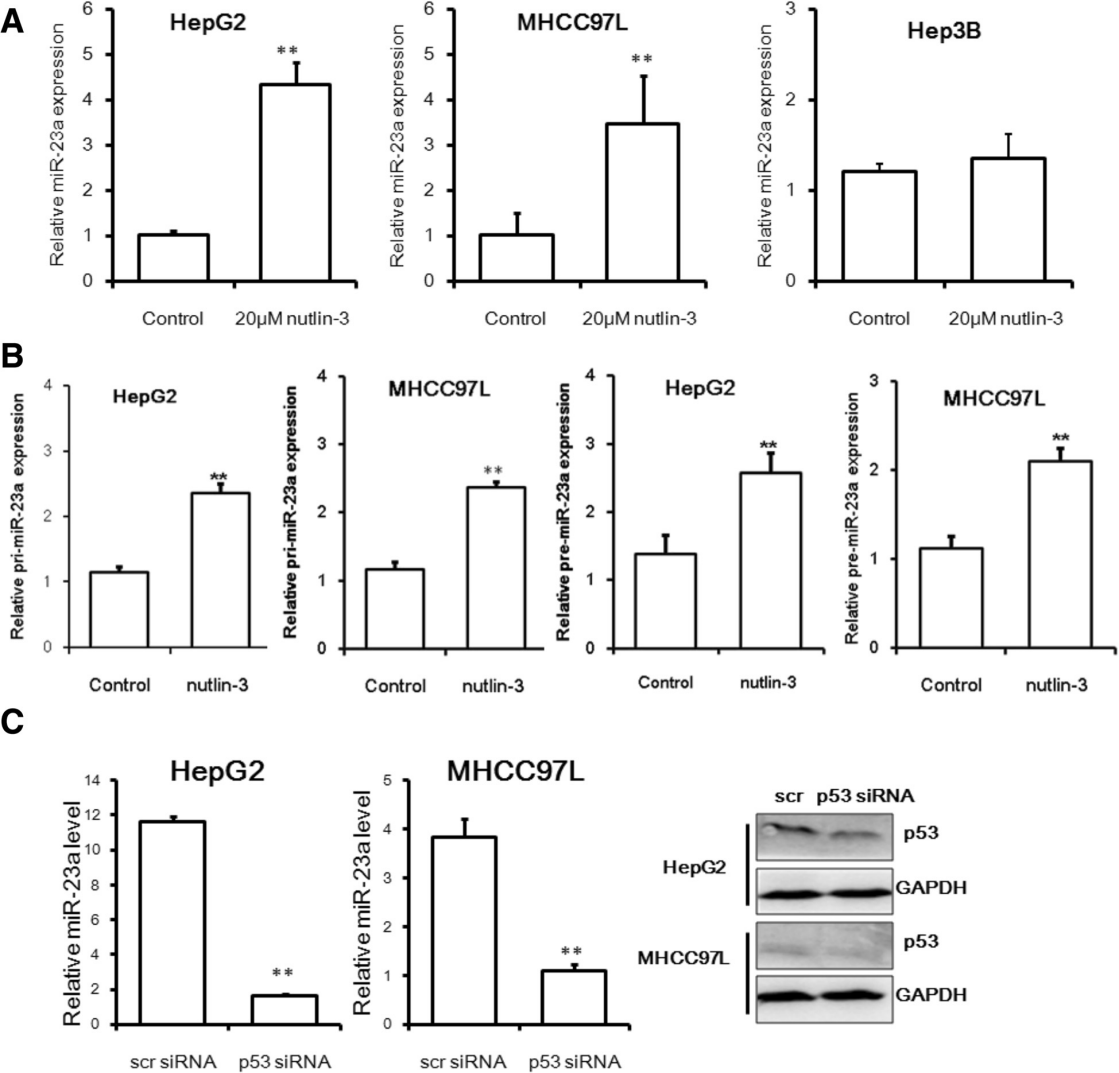
**Expression of miR-23a was control by p53. A** shows that induction of p53 by nutlin-3α up-regulates expression of miR-23a. HepG2, MHCC97L cells (p53-proficient) and Hep3B cells (p53-deficient) were treated with nutlin-3α (20 μM) for 24 h. The miR-23a expression was analyzed by qRT-PCR. Significant induction of miR-23a expression in HepG2 and MHCC97L but not Hep3B cells could be observed. **B** shows transcriptional activation of miR-23a induced by p53. Cells were exposed to nutlin-3α (20 μM) then the pri-miR-23a and pre-miR-23a expressions were analyzed by RT-qPCR; both primary and precursor form of miR-23a was up-regulated upon nutlin-3α treatment; **C** shows that suppression of p53 down-regulated miR-23a. Cells were introduced with RNAi against p53 and significant reduced expression of miR-23a could be observed in cells in which p53 was repressed.

p53 was reported to regulate miRNAs expression through various mechanisms [19,20]. It was most commonly observed that p53 acts as a positive and negative regulator of particular miRNA transcription [21,22]. Differential expression level of miR-23a could be found in the four HCC cell lines PLC/PRF/5, Hep3B, MHCC97L and HepG2, and the miR-23a level was correlated with p53 status in different HCC cell lines (Additional file 1: Figure S5). In p53-proficient cell lines, high level of miR-23a could be observed, but ablation miR-23a was found in Hep3B cells in which p53 is deficient. To further our knowledge on the regulation of miR-23a expression by p53, the MDM2 inhibitor nutlin-3α was used to activate p53 in HepG2 and MHCC97L. In both cell lines the transcription activity of p53 was induced by nutlin-3α, as evidenced by the increased expression of p53-downtream target genes upon nutlin-3α exposure (Additional file 1: Figure S6). Activation of miR-23a could be observed upon nutlin-3α treatment in p53-proficient HepG2 and MHCC97L cells, and it was observed that in p53-dificient Hep3B cells, nutlin-3α could not influence miR-23a expression (Figure 5A). The primary transcripts and the precursor forms of miR-23a were induced, indicating that p53 may transcriptionally up-regulate miR-23a expression (Figure 5B). Both RNA interference with siRNA against p53 and presence of inhibitor against p53 pifithrin-α could abrogate the expression of p53-downstreamed target genes (Additional file 1: Figure S7).The expression of miR-23a was suppressed when p53 is genetically or pharmacologically inhibited (Figure 5C and Additional file 1: Figure S8). These findings suggest the p53 is a positive regulator involved in miR-23a transcription activation.

## Discussion

The role of miR-23a in tumor progression is far from conclusive and still under investigation. Some previous studies showed that miR-23a could function to promote tumor progression by inducing tumor cell proliferation and invasion [3,23]. In our study, we found that expression of miR-23a may increase in HCC tissue when compared with non-tumor livers, which is consistent with some previous reports [15]. Mir-23a was showed to regulate the glucose metabolism during tumorigenesis via suppressing gluconeogenesis related genes. High levels of aerobic glycolysis are therefore induced, which lead to cancerous transformation of normal cells [15]. Moreover in our study, we found that miR-23a could target TOP1, which is essential to maintain genomic stability during DNA damage [24]. Loss of genomic stability once TOP1 is suppressed may therefore drive cancer development. However, as it was found that TOP1/TOP2A expression in cancer cells are important in determining the cellular response to chemotherapeutics. It was recently observed that inhibition of TOP1 level could potentiate response of tumor cells to doxorubicin treatment without altering the expression level of TOP2A [8]. Although the underlying mechanism remains not clear yet, it was shown in yeast that overall amount of topoisomerase activity is necessary for cell survival since more severe deficiencies of DNA unwinding, cell cycle progression and chromatin structure could be found in TOP1/TOP2A double mutant strains in compared to each single mutant [25,26]. It was therefore suggested that cells are vulnerable if the overall activity of topoisomerases is below the minimal level that cell itself sets [8]. Consistently it was observed in our study that TOP1 down-regulation by miR-23a in HCC cells could largely potentiate cells to etoposide and doxorubicin treatment but not 5-Fu exposure, and forced overexpression of miR-23a in HCC cells reduced the cytotoxicity of TOP1 poison irinotecan, whose activity was dominated by the content of endogenous TOP1 level [8]. The simultaneous TOP2A poisoning by etoposide with TOP1 inhibition results in cellular topoisomerase activity falling below the crucial threshold and triggering death of the cell. As etoposide is a TOP2A poison that induce covalent binding of TOP2A protein with DNA and results in DNA break and fragment in cancer cells, the etoposide sensitivity may be associated with the DNA repair mechanism in which the poly (ADP-ribose)polymerase (PARP) cleavage may be involved. Indeed, it was found that in etoposide-resistant cancer cells, deficiency of PARP cleavage was observed upon etoposide treatment [27]. It is probable that the compensatory activation of PARP cleavage may be initiated when TOP1 activity has also been suppressed in etoposide-treated cancer cells since previous study has found PARP cleavage in cells with TOP1 being inhibited [28]. As cleavage of PARP eventually results in cancer cell death, this compensatory action of TOP1 reduction in etoposide-treated cancer cell may therefore increase the chemotherapeutic sensitivity.

**Figure 6:**
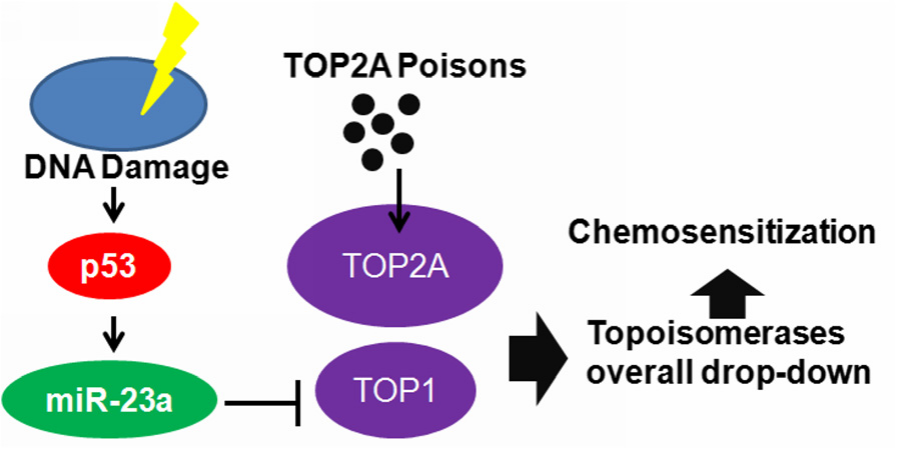
Overall regulatory mechanism underlying regulation of chemosensitivity by miR-23a in HCC cells.

A previous study has shown that presence of p53 may potentiate cellular response to etoposide treatment in cancer cells [29]. This may be correlated with p53-induced miR-23a expression, as it was indicated by our observation. p53 is well regarded as a tumor suppressive gene and is of aberrant ablation in human cancer cells. Inducible DNA damage is able to suppress the intracellular inhibitor of p53, and therefore up-regulates the p53 expression [30]. This may suggest the involvement of p53 in DNA damage-induced miR-23a expression in our observation. Some previous studies have found that inducing DNA damage by pharmacological approaches may enhance cellular response to etoposide treatment [31]. Our findings may advance the knowledge of DNA damage-induce etoposide sensitivity of cancer cells by introducing the involvement of miR-23a. Although literature also reports that high-dose of etoposide treatment could result in DNA damage [32], we could not observe potent genotoxic effect of etoposide in our study. HCC cells were highly resistant to common chemotherapeutics including etoposide and it is noticed that low dose of etoposide may not be enough to induce genotoxicity. However, our study may suggest the DNA damage-induced miR-23a is responsible for sensitizing HCC cells to etoposide treatment. Considering that direct overexpression of miR-23a in tumor cells may promote tumor progression as reported [33], it may be more practical to trigger miR-23a expression via inducing DNA damage by genotoxic agents, which may in consequence potentiate the response to etoposide treatment. A regimen in combining alkylating agent cisplatin with etoposide is commonly and traditionally in chemotherapy [34], in this regard, the findings of our study may add some experimental evidence in the use and optimization of etoposide-containing chemotherapeutic regimens. The involvement of miR-23a in potentiating cancer cells to etoposide treatment was shown in Figure 6.

## Conclusion

In closing, overexpression of miR-23a potentiates HCC cell to etoposide-induced cell death. Overexpression of miR-23a could not synergize 5-Fu-induced cytotoxicity and tumor inhibition, which indicates the mechanism of hypersensitivity induced by miR-23a is TOP2A poison-specific. Although TOP2A expression remained unchanged in miR-23a-overexpressing HCC cells, TOP1 was remarkably down-regulated, which may lead to the overall topoisomerase activity fall below the critical threshold for cell survival when cells are exposed to TOP2A poisons and consequently accelerates cell death. Both mRNA transcripts and protein expression of TOP1 are suppressed in miR-23a-overexpressing HCC cells, and luciferase assay shows that miR-23a may directly bind to the 3′UTR of TOP1 mRNA to suppress its expression. The interaction between miR-23a and TOP1 was further evidenced by the fact that forced overexpression of miR-23a could attenuate the cytotoxicity of TOP1 poison irinotecan. Activation of miR-23a could be induced during DNA damage in parallel with up-regulation of p53 expression. Activation of p53 could increase the transcripts of pri-, pre- and mature form of miR-23a, while inhibition of p53 significantly reduced miR-23a level in p53-proficient HCC cells, indicating that miR-23a may be transactivated by p53 in HCC cells. Our study shed lights on the potential of miR-23a as a novel target in regulating chemosensitivity of cancer cells.

## Competing interests

The authors declare that they have no competing interests.

## Authors’ contributions

YF designed the experiment, interpreted the data and prepared the manuscript. NW and MZ conducted the experiment, collected the data and helped to prepare the manuscript. SWT, KM and ZZ interpreted the data. All authors read and approved the final manuscript.

## Supplementary Material

Additional file 1 Supplemental data. Click here for file
