# Supplementary material for: MiR-23a-mediated inhibition of topoisomerase 1 expression potentiates cell response to etoposide in human hepatocellular carcinoma
Source: Mol Cancer. 2013 Oct 8;12:119. doi: 10.1186/1476-4598-12-119 (PMC3856574; doi:10.1186/1476-4598-12-119)
Supplement: Additional file 1 — Supplemental data. [file 1476-4598-12-119-S1.docx]

***Supplemental Data***

**Fig.S1 Expression of miR-23a in human hepatocellular carcinoma tissue**

All RNAs were collected by Origene from 3 non-tumor and 15 HCC tissues. Quantitative analysis on miR-23a expression was conducted. The results shows that increased expression in HCC samples, however, we cannot see statistical difference between normal group and HCC group (p=0.15).

**Fig. S2 ectopic expression of miR-23a has no effect on the impairment of HCC cell cycle induced by 5-Fu**

Overexpression of miR-23a has no effect on 5-Fu-induced cell cycle impairment in HCC cells. Wildtype and miR-23a-overexpressed HCC cells were treated with etoposide (50 μg/mL) for 24 h and then fixed. Cells were then stained with PI for cell cycle analysis. It was found that 5-Fu may alter the cell cycle distribution in HCC cells, however, overexpression of miR-23a in HCC cells has no effect on the impairment of HCC cell cycle induced by 5-Fu treatment.

**Fig.S3 miR-23a was induced in HCC cells with hydrogenperoxide treatment**

Cells received 1 mM H_2_O_2_ treatment for 24 h. Total RNA was collected and miR-23a was detected by RT-qPCR. H_2_O_2_ treatment significantly induce miR-23a expression in HCC cells.

**
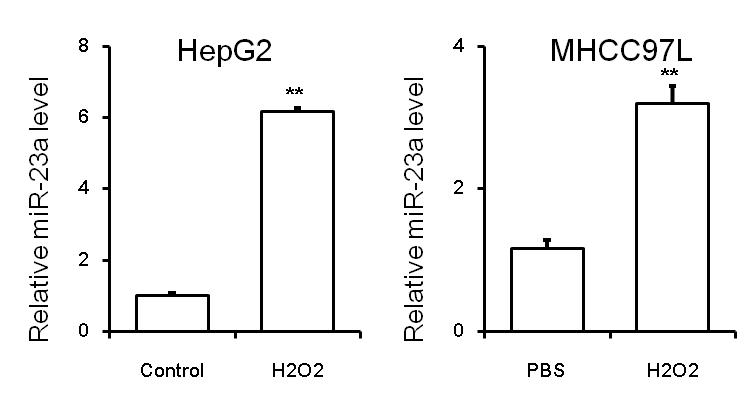
**

**Fig. S4 DNA damage induces expression of p53 in HCC cells.**

Cells were seeded into 6 well plates and received UV-C treatment for 10 minutes and incubated for 24 h. Protein was then collected and analyzed.

**Fig.S5 Expression of miR-23a was correlated with p53 status in HCC cells.**

Positive correlation of miR-23a expression with p53 level could be observed in HCC cell lines.

**Fig.S6 Expression of p53-downtreamed target genes was increased upon nutlin-3α**

Cells were treated with nutlin-3α (20 μM) for 24 hr and RNA was collected. The expression of p53 downstreamed target genes, p21, GADD45α, TP53INP1 was detected by qRT-PCR. Induction of gene expression by nutlin-3α could be observed.

**Fig.S7 Treatment of pifithrin-α reduces p53 transcription activity.**

Cells were treated with pifithrin-α (50 μM) for 24 hr and RNA was collected. The expression of p53 downstreamed target genes, p21, GADD45α, TP53INP1 was detected by qRT-PCR. Reduced gene expression could be observed in pifithrin-α-treated cells.

**Fig. S8 Pharmacological inhibition of p53 suppresses miR-23a expression.**

Cells were treated with p53 inhibitor pifithrin-α (50 μM) and potent reduced expression of miR-23a could be observed.
